# Male Breast Cancer With Dual BRCA2 and BRIP1 Deleterious Gene Mutations

**DOI:** 10.31486/toj.23.0119

**Published:** 2024

**Authors:** Shivani B. Badve, Emily Kim, Udai S. Sibia, Orestes T. Borrego, Stephen Vara, Alexander Damron, Adam I. Riker

**Affiliations:** ^1^ Department of Surgery, Luminis Health, Annapolis, MD; ^2^ Loyola University Chicago, Chicago, IL; ^3^ Saint John's Cancer Institute, Santa Monica, CA; ^4^ Department of Pathology, AmeriPath, Fort Myers, FL; ^5^ Department of Medical Oncology, Florida Cancer Specialists & Research Institute, Port Charlotte, FL; ^6^ Precision Healthcare Specialists, Naples FL

**Keywords:** *Breast neoplasms–male*, *genes–BRCA2*, *genes–neoplasm*

## Abstract

**Background:** Male breast cancer remains relatively underexplored in the medical literature. At present, male patients with breast cancer follow the same treatment guidelines as female patients with breast cancer, principally because of similar outcomes with treatment. However, this practice should not preclude generating evidence for male breast cancer surveillance, diagnosis, and management. BRCA2 gene mutations are associated with an increased risk of male breast cancer, along with lesser-known gene mutations that could also increase this risk, such as mutations of the BRIP1 gene. This case report presents a male patient with dual BRCA2 and BRIP1 deleterious gene mutations. To our knowledge, this combination has not been reported in the medical literature to date.

**Case Report:** A 53-year-old male presented with a palpable symptomatic mass underneath the right nipple-areolar complex. Biopsies confirmed a poorly differentiated, infiltrating ductal carcinoma that was estrogen and progesterone receptor positive and human epidermal growth factor receptor-2 negative. The patient underwent a left modified radical mastectomy, with a right prophylactic simple mastectomy. Postoperatively, he underwent adjuvant chemotherapy and endocrine therapy.

**Conclusion:** This novel case of genetically based male breast cancer with dual deleterious gene mutations provides insight into current treatment recommendations and the subtle differences between male breast cancer and female breast cancer. Engaging in discussions surrounding such rare cases not only raises awareness of male breast cancer but also indicates the need for further research aimed at establishing evidence-based management strategies for male patients with breast cancer.

## INTRODUCTION

Although males represent <1% of those diagnosed with breast cancer, the overall mortality rate for males with breast cancer is significantly higher than that of women.^1,2^ This difference could be attributable to our evolving understanding of breast cancer in males, including biomarkers and optimal management strategies. Male breast cancer research is scarce, partly because of the lower incidence of breast cancer in males and the assumption that findings from female breast cancer studies can be applied to managing male breast cancer.

In this case, we describe the management of a male breast cancer patient with both BRCA2 and BRIP1 deleterious gene mutations. BRIP1 stands for BRCA1 interacting protein 1, otherwise known as BACH1 (BRCA1-associated C-terminal helicase 1). BRIP1 has been associated with an increased risk for breast cancer but is quite rare.^3^ Studies have concluded that the effect of BRIP1 on the risk of developing breast cancer may be negligible, but it is still commonly tested for as no robust evidence is available on the clinical significance of such a small population. To the best of our knowledge, this case is the first reported instance of a male patient with breast cancer who has both gene mutations.

## CASE REPORT

A 53-year-old otherwise healthy male presented to his primary care physician for evaluation of a palpable left breast mass that he had first noticed several months prior. He stated that the mass was slowly growing and had recently started causing pain and discomfort. The patient also complained of his left nipple inverting. He felt as if the skin around his left nipple was getting thicker, and he recently felt a lump in his left axilla. He denied Ashkenazi Jewish heritage and any personal history of cancer or radiation. His family history was notable for breast cancer in his paternal aunt in her mid-40s.

**Figure 1.**
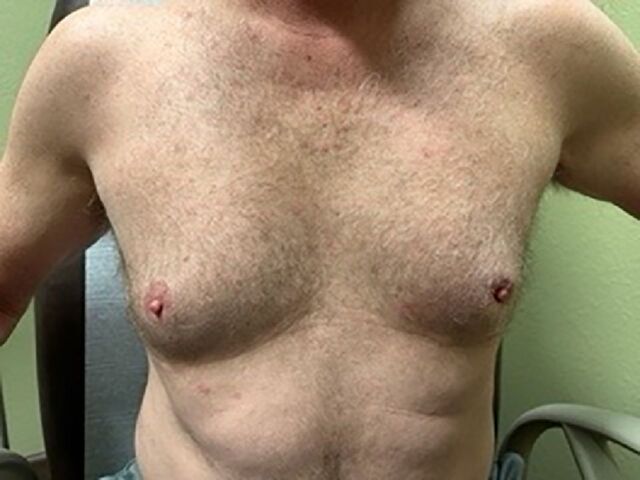
At the patient's initial clinic visit, the mass was palpable in the left breast under the nipple-areolar complex.

On examination, the patient had a subareolar 2-cm hard mass within the left breast with slight left nipple inversion (Figure 1) and an associated 3-cm palpable lymph node in the level II region of the left axilla. There was no associated nipple discharge.

Diagnostic mammogram and left breast and axillary ultrasound revealed a suspicious mass (Breast Imaging Reporting and Data System [BI-RADS] category 4 lesion) within the subareolar region of the left breast that measured 2 cm in greatest diameter. Left axillary ultrasound identified a 4-cm suspicious-appearing lymph node.

**Figure 2.**
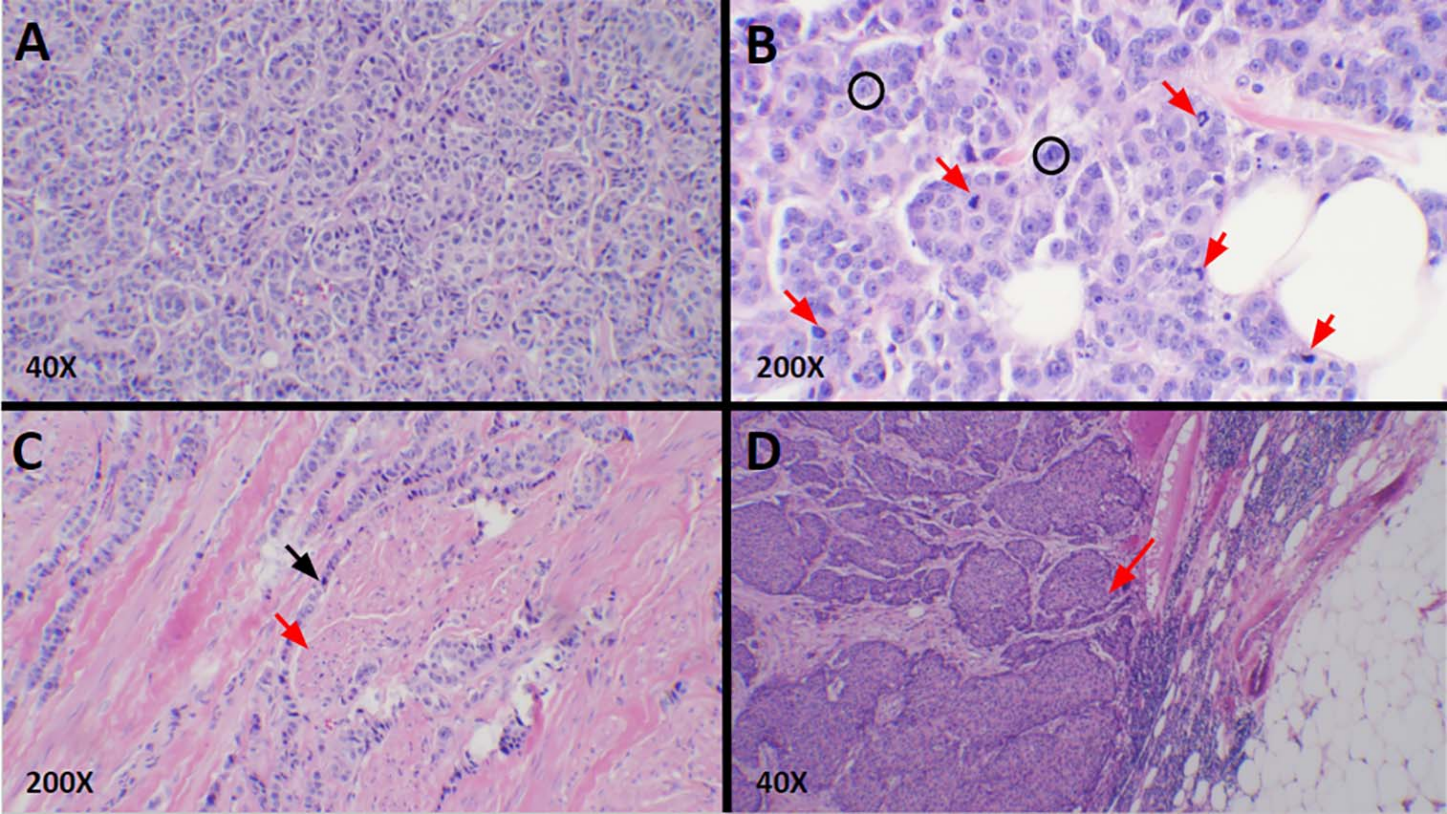
Hematoxylin and eosin–stained sections from patient pathology. (A) Infiltrating ductal carcinoma had a predominant solid sheet-like and nested growth pattern lacking tubule formation. No significant lymphocytic infiltrate was associated with the invasive carcinoma. (B) Frequent mitotic activity was noted (red arrows). The tumor showed up to 18 mitoses per 10 high-power fields and also displayed grade 2 to 3 nuclear pleomorphism with cancer cells of varying sizes and shapes displaying prominent nucleoli and irregularly shaped nuclei (black circles). The tumor was classified as Nottingham grade 3. (C) Infiltrating ductal carcinoma (black arrow) surrounded the erector pili muscles (red arrow) of the nipple dermis. There was no epidermal involvement and no associated ulceration. The dermal invasion did not impact the American Joint Committee on Cancer pathologic T stage of this cancer. (D) Axillary lymph node showed metastatic carcinoma (red arrow) with a similar morphology to the tumor cells in the breast, consistent with metastasis from the patient's known breast primary.

**Figure 3.**
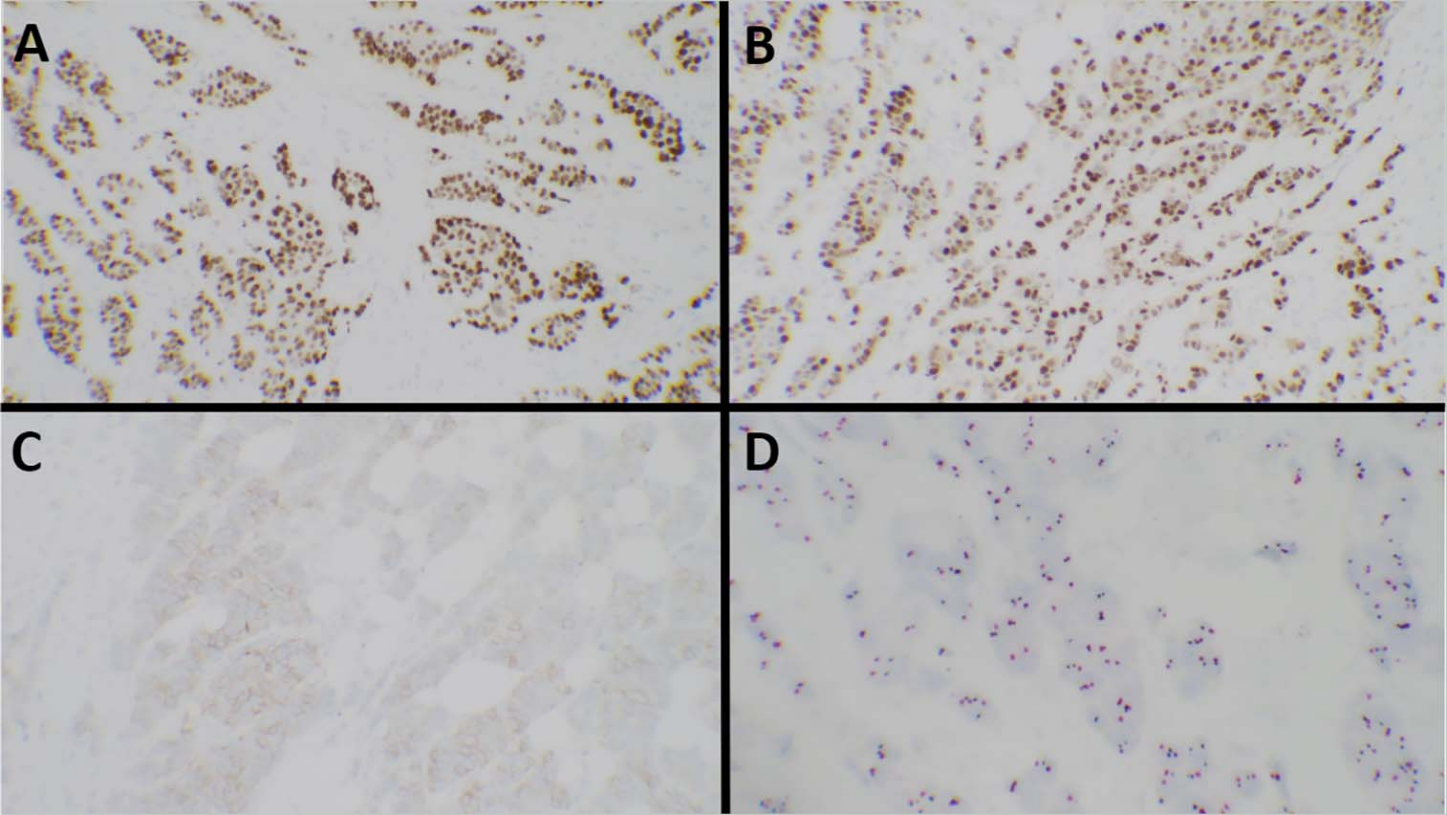
(A) Biomarker studies showed the tumor cells to be strongly positive for estrogen receptor protein in 100% of the tumor cells and (B) progesterone receptor protein in 95% of the tumor cells. (C) Human epidermal growth factor receptor-2 (HER2) immunohistochemistry showed weak (score 1+) staining with moderate (2+) staining in <10% of the tumor cells. (D) HER2 in situ hybridization showed negative (nonamplified) results with a HER2/CEP17 (chromosome enumeration probe 17) ratio of 0.9.

Core needle biopsy of the left breast mass was performed. Pathology revealed a poorly differentiated infiltrating ductal carcinoma, estrogen receptor positive (ER+) 100%, progesterone receptor positive (PR+) 95%, and human epidermal growth factor receptor-2 (HER2) negative. Immunohistochemical staining was 1+ for HER2 (Figures 2 and 3). A 47-gene panel of common hereditary cancers was performed, and 2 pathogenic variants were identified in the BRCA2 and BRIP1 genes. A third variant of unknown clinical significance was identified in the CTNNA1 gene.

Genetic counseling and testing were performed, utilizing a sequence analysis and deletion/duplication of a panel of 47 genes, including analysis of the BRCA1 and BRCA2 genes (Invitae Common Hereditary Cancers Panel, BRCA1/2 Panel, Invitae Corporation). The results revealed pathogenic, heterozygous variants [c.9294C>G (p.Tyr3098*)] in the BRCA2 gene and in the BRIP1 gene [c.1871C>A (p.Ser624*)], with a third variant of unknown clinical significance in the CTNNA1 gene [c.2633C>G (p.Thr878Arg)].

Following the core biopsy, the patient was evaluated and seen by a medical oncologist who referred the patient for surgical consultation.

After a multidisciplinary team discussion, the patient elected to proceed with a left modified radical mastectomy and prophylactic right simple mastectomy for symmetry on April 7, 2023. The patient had no intraoperative or postoperative complications.

Final pathology demonstrated a 25-mm poorly differentiated infiltrating ductal carcinoma, grade 3, involving the dermis and skin. All final surgical margins showed no ink on tumor. The closest margin measured 8 mm posteriorly. A total of 18 left axillary lymph nodes were removed, with 2 of the 18 nodes positive for metastatic disease. The largest nodal metastatic deposit measured 16 mm and showed evidence of extranodal extension in both tumor-positive lymph nodes.

Genomic profiling with Oncotype DX (Exact Sciences Corporation) revealed a recurrence score of 31. This result correlated with a high risk of distant recurrence at 9 years with an aromatase inhibitor or tamoxifen alone of 24% and an absolute overall survival benefit with chemotherapy of approximately 15%. Adjuvant systemic chemotherapy was recommended with the plan for dose-dense doxorubicin and cyclophosphamide, followed by paclitaxel. Based on the results of the monarchE trial,^4^ adjuvant endocrine therapy with tamoxifen was also recommended.

Breast cancer is a known familial cancer, with the BRCA2 gene identified as an autosomal dominant inheritance pattern without 100% penetrance.^5^ The presence of a BRCA2 mutation in this patient indicated the possibility of an increased risk of breast, ovarian, pancreatic, and prostate cancer in other at-risk family members. Genetic testing of several family members revealed the same deleterious gene mutations present in his mother and daughter. The patient's son tested negative for the mutations.

The patient continues to undergo adjuvant treatment. He was referred to radiation oncology with the recommendation to receive postmastectomy left chest wall and axillary radiation therapy after completing his adjuvant chemotherapy to decrease the risk of a locoregional recurrence. Despite counseling regarding the benefits of radiation, the patient refused this treatment as he was concerned about toxicity. The patient is followed every 6 months by his medical and surgical oncologists.

## DISCUSSION

Specific genetic mutations are associated with an increased risk of breast cancer. The risk of developing breast cancer is increased by 7% to 8% in males with the BRCA2 mutation, compared to a 1% risk for males with BRCA1.^6,7^ BRCA2 mutations are the most common in male breast cancer; however, this mutation is not the only mutation that is tested for.^8^

Seal et al concluded that monoallelic BRIP1 mutations can confer an increased susceptibility to breast cancer compared to the general population.^3^ BRIP1 is a helicase that is involved in DNA repair functions of BRCA1.^9,10^ Experimental data conclude that in cases of a BRCA1 mutation, the BRIP1 protein is unable to function properly. The subsequent accumulation of mutations can then become resistant to chemotherapy-induced apoptosis.^10^ Studies published after the Seal et al article (2006) have associated BRIP1 mutations with a higher risk of ovarian cancer than breast cancer.^11-13^ Patients with breast cancer are still tested for BRIP1 mutations but with a very low suspicion of a positive result. No data specifically connect BRIP1 mutations with BRCA2 breast cancer.

In terms of treatment, a common difference in management is noted in early-stage breast cancer. Because of their limited amount of breast tissue, males can opt for simple mastectomy rather than breast-conserving therapy. Beyond that, the decision-making process mirrors that of females. For those with hormone receptor–positive disease, as in our patient, the current recommendations support tamoxifen adjuvant therapy for 5 years, barring any contraindications.^14^ Chemotherapy and radiation therapy can be administered based on the same indications as for females because no robust evidence currently suggests otherwise.^15^

Regarding hormone receptors, male patients with breast cancer have a higher proportion of ER and PR positivity and a lower proportion of HER2 positivity compared to female patients, which are findings consistent with our case.^1,16^ Male breast cancer patients with the hormone receptor–positive/HER2-negative subtype have been shown to have worse overall survival compared to stage- and subtype-matched female breast cancer patients.^2,17^

The unprecedented nature of this case raises several questions that merit further research. Whether these 2 genes are additive in risk or coexist as independent risk factors is unclear. Furthermore, because BRIP1 also confers a risk of ovarian cancer,^11^ exploring the relative significance of a BRIP1 mutation in males vs females would be interesting.

## CONCLUSION

To our knowledge, the presence of both BRCA2 and BRIP1 deleterious mutations has not been identified in the literature, especially in male breast cancer. As cancer detection technologies improve and the awareness of male breast cancer increases, this report contributes to an evolving understanding of male breast cancer management, especially in unique cases such as this.

## Notes

*This article meets the Accreditation Council for Graduate Medical Education and the American Board of Medical Specialties Maintenance of Certification competencies for Patient Care and Medical Knowledge.*
